# Investigation of the ionic conditions in SiRNA-mediated delivery through its carriers in the cell membrane: a molecular dynamic simulation

**DOI:** 10.1038/s41598-022-22509-1

**Published:** 2022-10-20

**Authors:** Mohammad Hasan Darvishi, Abdollah Allahverdi, Hadi Hashemzadeh, Hamid Reza Javadi

**Affiliations:** 1 Nanobiotechnology Research Center, Baqiyatallah University of Medical Sciences, Tehran, Iran; 2 Department of Biophysics, Faculty of Biological Science, Tarbiat Modares University, Tehran, 14115-154 Iran

**Keywords:** Biophysics, Computational biology and bioinformatics

## Abstract

SiRNA is a new generation of drug molecules and a new approach for treating a variety of diseases such as cancer and viral infections. SiRNA delivery to cells and translocation into cytoplasm are the main challenges in the clinical application of siRNA. Lipid carriers are one of the most successful carriers for siRNA delivery. In this study, we investigated the interaction of siRNA with a zwitterionic bilayer and how ion concentration and lipid conjugation can affect it. The divalent cation such as Mg^2+^ ions could promote the siRNA adsorption on the bilayer surface. The cation ions can bind to the head groups of lipids and the grooves of siRNA molecules and form bridges between the siRNA and bilayer surface. Our findings demonstrated the bridges formed by divalent ions could facilitate the attachment of siRNA to the membrane surface. We showed that the divalent cations can regulate the bridging-driven membrane attachment and it seems the result of this modulation can be used for designing biomimetic devices. In the following, we examined the effect of cations on the interaction between siRNA modified by cholesterol and the membrane surface. Our MD simulations showed that in the presence of Mg^2+^, the electrostatic and vdW energy between the membrane and siRNA were higher compared to those in the presence of NA^+^. We showed that the electrostatic interaction between membrane and siRNA cannot be facilitated only by cholesterol conjugated. Indeed, cations are essential to create coulomb repulsion and enable membrane attachment. This study provides important insight into liposome carriers for siRNA delivery and could help us in the development of siRNA-based therapeutics. Due to the coronavirus pandemic outbreak, these results may shed light on the new approach for treating these diseases and their molecular details.

## Introduction

The small interfering RNA (siRNA) is a group of small non-coding RNA that has roles in a wide range of cellular processes^1^. The RNA interference pathway involves post-transcriptional inhibition of complementary messenger RNAs (mRNA) and silence-related gens^2,3^. The siRNA-based therapy has been interested as a new approach for treating diseases, such as cancer diseases and virus infection^4–6^. The siRNA-based therapy is currently in the final stages of clinical trials^7,8^. The siRNA-based therapy has several limitations, such as low stability and poor cellular uptake properties^9^. The new approaches to overcome these limitations are nonviral delivery systems, including liposomes, inorganic nanoparticles, and carbon nanomaterials^10,11^.

Coronaviruses are one of the most important issues of public health in the world. The respiratory syndrome coronvirus1 outbreak in 2003 and the Middle East respiratory coronavirus outbreak in 2012 infected approximately 8096 cases. The novel coronavirus SARS-Cov2 presents with a wide range of symptoms, which the main developing symptoms involve multi-organ failure, and acute respiratory distress syndrome^6^. In the wake of the pandemic breakout, several coronavirus vaccines have developed. However, the SARS-CoV-2 therapies are essential to treat coronavirus disease in 2019^12,13^. During the pandemic, several siRNAs have been designed to target the conserved coronavirus region of SARS-CoV-2^14–17^. In addition, the siRNA with lipid nanoparticles can improve the inhibition of virus expression in vivo conditions^6^.

Therefore, siRNA molecules are the new generation of drug molecules and have high efficiency in clinical trials^18,19^. The delivery of siRNA to cells and translocation into cytoplasm are the main challenges in the clinical application of siRNA^20,21^. The siRNA molecules attach to the negative surface of the bilayer, and after, that cross through the cell membrane and enter the cytosol^1^. Crossing siRNA through the hydrophobic region of the bilayer is the main challenge in siRNA delivery^22,23^. The naked siRNA is unstable and rapidly degraded by serum nuclei. It seems, that the preservation of the siRNA from enzymatic degradation and low permeability of siRNA in bilayer systems are the main challenges in siRNA application in clinical trials^24,25^. The delivery systems are the primary solutions for these challenges. There are several approaches to enhance the delivery of nucleic acids such as electric pulse, hydrodynamic injection, and siRNA delivery carriers^26,27^. For siRNA delivery, several delivery carriers exist, which preserve the siRNA from degradation and transfer across the cell membrane. A wide range of carriers was used in siRNA delivery such as liposomes, cationic polymers, and conjugation of siRNA to a ligand such as cholesterol^19,28–31^. In a chemical modification, cholesterol-siRNA conjugation is a highly efficient system in siRNA delivery. Cholesterol is a known carrier in the nucleic acid delivery system^32,33^. Several studies showed that the ionic concentration could improve the attachment of siRNA on the membrane surface^34–36^. The efficiency of siRNA delivery of Chol-siRNA conjugated in the presence of an ionic concentration of divalent cations was higher than Chol-siRNA in the absence of divalent. It seems the lipid modification of siRNA is not sufficient for nucleic acid–lipid adhesion and the ionic concentration required for siRNA attachment to the membrane surface^35^.

Liposomes are the most widely used delivery systems for the siRNA molecules^19^. Previous studies show that liposomes with cationic lipids are the most effective delivery systems for siRNA delivery and improve the cellular uptake properties^5,19^. However, the high toxicity of these delivery systems restricts the use of these lipid-based delivery systems in clinical trials. Some neutral lipids such as DOPC lipids show higher efficiency in siRNA delivery compared to cationic lipids (DOTAP lipids)^19,37^. Therefore, the interaction of siRNA with zwitterion lipids is a key parameter in the siRNA delivery systems. Zwitterion (neutral) lipids are one of the safer alternatives that could be used instead of cationic lipids. The neutral lipids are used as helper lipids, which can improve liposome stability. Recent studies show that siRNA delivery systems based on Zwitterion liposomes have higher antitumor efficiency compared to cationic-liposome delivery systems. Previous studies showed that the attractive interactions between DNA and lipid can be modulated by divalent cations such as calcium and magnesium. Since siRNA is a polyanionic molecule, it seems the divalent cation can have similar effects on siRNA-lipid interactions. The adsorption of siRNA on the bilayer can lead to the formation of a stable siRNA-lipid structure^38–41^.

Therefore, the interaction of siRNA with Zwitterion-lipids is a critical player in the development of siRNA-based systems and siRNA therapies^19,37^. The interaction of siRNA with Zwitterion-lipids is related to a wide range of parameters such as ionic condition, lipid shape, and cholesterol concentration^39,40^.

The stable siRNA-lipid complex could play a critical role in delivery systems. For example, the positively charged groups of lipids can adsorb the phosphate groups of DNA molecules and form electrostatic interactions between two groups. This charge surface promotes the efficient delivery of DNA and cellular internalization^42^. Mengistu et al. indicated that divalent cations such as calcium, magnesium, and others could promote the interactions between DNA and membrane. This ability of divalent cations can help us to design lipid based gene delivery systems^43^.

In the following, the ionic condition is a critical parameter in siRNA adsorption on the lipid surface^39^. It seems the ionic condition can modulate the interaction siRNA lipid system^34^. In addition, previous studies showed that divalent cations such as magnesium and calcium could improve the attraction of siRNA to bilayer systems^4^. It seems that the ionic condition could promote the interaction of siRNA-lipids. Despite the important role of zwitterion lipids in siRNA delivery systems, the siRNA-lipid interactions are not fully understood. In addition, a variety of parameters can affect these interactions, which are not clear. A siRNA molecule is a short double-stranded with two unpaired nucleotides on each end. The small size of siRNA makes it the best object to study the siRNA-lipids by atomistic molecular dynamic simulations^34^. In previous studies, molecular dynamics simulations were carried out to define the atomic details of the interactions between siRNA and cationic polymer carrier systems. However, there are few studies about the molecular details of siRNA–lipid interactions. The phospholipid molecules are an essential part of delivery systems such as liposome systems and lipid conjugation systems. Tarek and coworkers^44^ investigated the translation of siRNA across the membrane by applying an external electric field. It has been demonstrated that the siRNA–lipid interaction was preserved after the electric field was switched off. These findings show that the interaction of siRNA and the head group of lipids an essential role in the adsorption of siRNA on the membrane surface. In another study, the efficiency of the different carriers was investigated by molecular dynamic simulation. It has been reported that the lipid conjugation systems could improve the efficiency of the siRNA delivery system^30^. In addition, between PAMAM (Poly amidoamine) and cholesterol as a carrier, cholesterol is a more efficient carrier for siRNA delivery than PAMAM^45^. The zwitterion lipids with low toxicity can be used as an alternative lipid delivery system. The neutral lipids are often used as helper lipids to increase liposome stability^3^.

**Figure 1:**
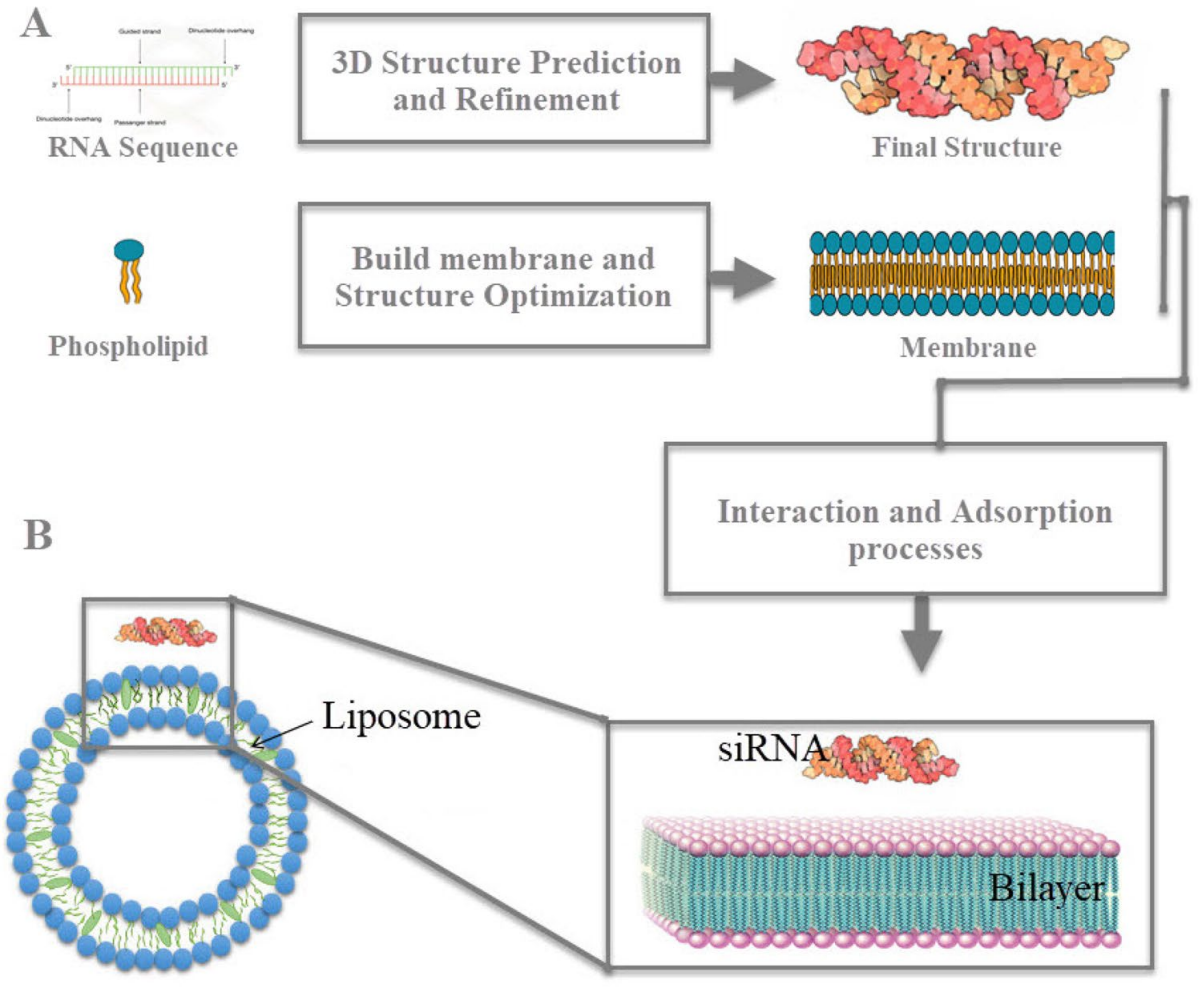
(**A**) The structure prediction and refinement of siRNA and the building membrane, (**B**) Schematic representation of the liposome-RNA complex and the bilayer—RNA system, which is a model for studying siRNA adsorption on the membrane surface.

In the present study, the molecular details of the mechanism of siRNA with zwitterion lipids interaction and the effect of salt conditions on this interaction were analyzed. For this goal, atomistic molecular dynamic simulations were applied to get a detailed insight into the siRNA–lipids interaction in molecular detail as shown in Fig. 1A,B. Here, we showed that the ions could improve the electrostatic interaction between nucleic acids and lipids. Our findings demonstrate that the cations can increase the interaction of DNA—lipid. In addition, the cations can change the physical properties of the bilayer, such as thickness and fluidity.

## Methodology

### Membrane preparation

We have carried out molecular dynamic simulations of siRNA in the aqueous solution and after that, the siRNA was placed near the POPC membrane as a zwitterion membrane. The siRNA was oriented parallel to the bilayer. The distance between siRNA and the bilayer in the initial structure was defined as 1.2 nm. The different concentrations of monovalent and divalent salt conditions were applied in simulations.

The simulation box included a siRNA molecule and a bilayer membrane model of 400 POPC lipids. The membrane was solvated with the TIP3P water molecules. The magnesium and chloride ions were added to the system at 100 mM concentration. The concentration of MgCl_2_ and NaCl were chosen based on previous studies^34,35^. All of the systems were minimized by the conjugated gradient methods to remove all bad contacts and steric clashes. In the following, the systems were equilibrated at a constant of the number of atoms, the pressure of 1 bar, and temperature (T = 303 K). The siRNA consists of a 20 pair-based double-strand and two overhanging unpaired nucleotides. The initial structure of siRNA was obtained from the w3DNA server (http://web.x3dna.org/fibermodel/rnaseq)^46^. The double strands section of siRNA is generated based on the A-DNA.

The force field of siRNA was obtained from the Charmm36 force field^47^. The bilayer of POPC lipids was generated by the CHARMM-GUI server (https://charmm-gui.org). All simulations were carried out by Gromacs 2020 and the Charmm36 force field was used^48^. The box of the simulation was solvated by the TIP3P water model, and standard parameters of CHARMM were used for ions.

### Cholesterol-siRNA conjugation

Cholesterol is used as a hydrophobic carrier for siRNA delivery and investigates the effect of cholesterol on the siRNA–lipid interaction. The initial 3D structure of cholesterol was built in the CHARMM GUI membrane builder^49^. After that, the cholesterol was bound to the 5’ terminal of siRNA. The 3D structure of siRNA-Cholesterol was optimized. The force field of siRNA–cholesterol conjugation was built by CGenFF^50^.

The systems were minimized using the steepest descent algorithm. All simulations were carried out in an NpT ensemble at T = 303 K and P = 1 bar. The periodic boundary conditions were applied in all directions. The PME method was used in long-range interactions and a cutoff of 1.2 nm was used for vdW (Van der Waals) interactions calculation. In the equilibration section, the velocity-rescaling thermostat was used to control temperature and the Berendsen Barostat was used to control pressure in anisotropic conditions. The cut-off of Lennard–Jones interactions was 1.2 nm. The particle mesh Ewald was applied to calculate long-range interactions. The time step was 2 fs.

### Equilibration and production procedure

The membrane and siRNA molecule were added to the simulation box and were equilibrated by multi-step methods. The first step contained the steepest descent by position-restrained MD with harmonic restrained on all non-hydrogen atoms in the simulation box. All MD simulation and minimization processes were applied by the Gromacs program and CHARMM force field generator. All simulations were equilibrated by the NVT and NPT ensembles. The temperature and pressure were kept to 303 K and 1 atm. The anisotrophic Berendsen barostat was used for pressure control. All bonds containing hydrogen and non-hydrogen bonds were constrained by the SHAKE algorithm. The Particle Mesh Ewald was used for the calculation of short-range electrostatics. The integration time step of 2 fs was used. The structures, velocity, and energies of systems were saved every 100 picoseconds. The Gromacs package was used for the analysis of the trajectories of MD simulations.

#### Area per lipid

This parameter is important in describing the physical parameters of the membrane systems. In MD simulation systems, the lipid bilayer is orientated in the z direction of the simulation box. The APL was calculated by the following equation:

$$ {\text{APL }} = {\text{ L}}_{{\text{x}}} {\text{L}}_{{\text{y}}} /{\text{N}}_{{{\text{lipid}}}} , $$

L_x_, L_y_ indicate the box length in x and y direction. N shows the number of lipids in one leaflet.

The thickness of the membrane was calculated by the distance between the phosphate groups of the upper and lower leaflets. The radial distribution functions were calculated by observing the distances between phosphate head groups of lipids belonging to the center of mass siRNA molecule.

### Data analysis

The electrostatic and vdW energy and distances were calculated by the Gromacs package. In the analysis of the membrane, the membrane properties such as the order parameter, lipid per area, and thickness of the membrane were calculated using the analysis section of the Gromacs program. Furthermore, the lateral distribution of Mg ions in the box of simulations, RDF between siRNA and head groups of lipids, the number of contacts between siRNA and the membrane, and the orientation of siRNA molecules related to the z-direction of the membrane were measured by tools in the VMD and Gromacs packages.

## Results and discussion

### The effect of divalent ions on the adsorption of siRNA on the bilayer

**Figure 2:**
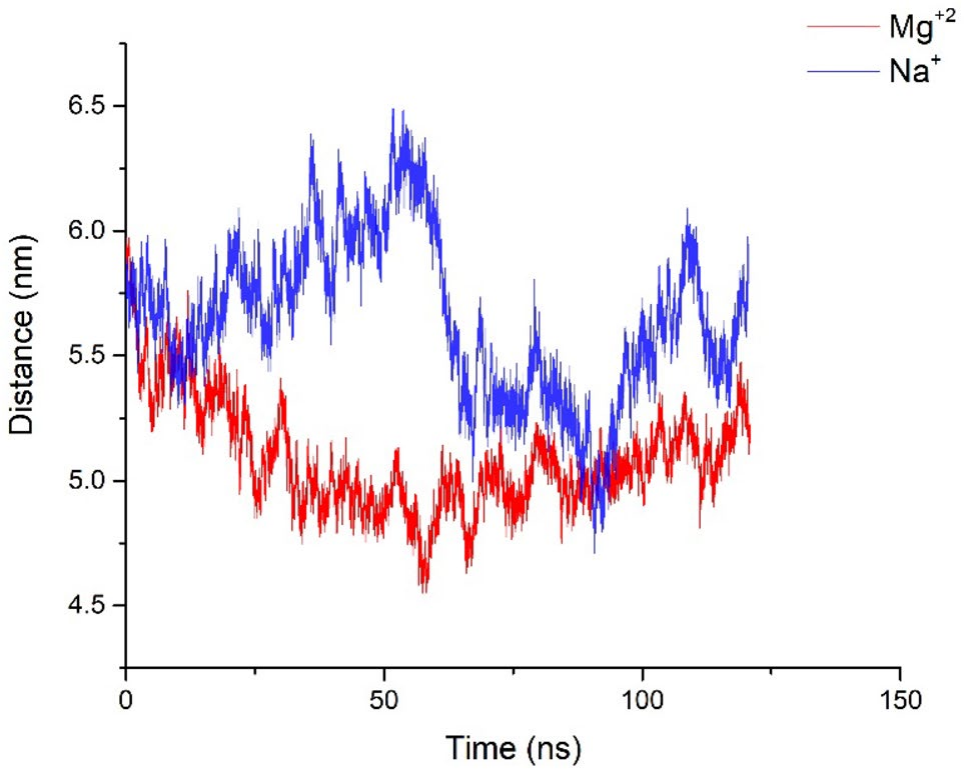
The distance between the center of mass bilayer and siRNA in the absence and presence of Mg^2+^ ions.

To observe the adsorption of siRNA on the lipid bilayer, siRNA was first placed parallel to the bilayer. We carried out four MD simulations. To measure the adsorption of siRNA on the bilayer, the distance between the center of mass bilayer and siRNA was measured. The mean distance between the center of mass siRNA and the membrane in the presence and absence of Mg^2+^ are 5.1 and 5.6 nm. Firstly, in all systems, the siRNA can be adsorbed on the bilayer surface, although these interactions are unstable as shown in Fig. 2. However, the presence of divalent cations decreased the distance between siRNA lipids compared to monovalent cations as shown in Fig. 2.

Previous results showed that in the presence of divalent cations, DNA does not adsorb on the surface of the neutral membrane^35,39^. In addition, it has been reported that DNA adsorption on the membrane surface is a barrier energy in crossing DNA through the membrane^35^. The siRNA has unpaired nucleotides at two ends. It seems the charge of these ends could modulate the interaction between siRNA and the head group of the membrane surface. Further, the conformation of siRNA (A-from) is different from DNA (B-form) molecule. The conformation B-form has wider major grooves and narrower minor grooves. These grooves are suitable positions for nucleic acid—ions interactions. Mirsa et al. demonstrated that the divalent ions such as magnesium could bind to site-bound ions on tRNA and rRNA molecules and these events can play an important role in RNA stability^51^. In a different study, Laing et al. showed that magnesium ions can bind to RNA in different modes. The hairpin structures were stabilized by non-specific binding. The tertiary structure of RNA, such as ribosomal RNA, is stabilized by specific binding of Mg^2+^. In the third class of sites, the divalent ions can be placed in a region with a high charge density^52^.

**Figure 3:**
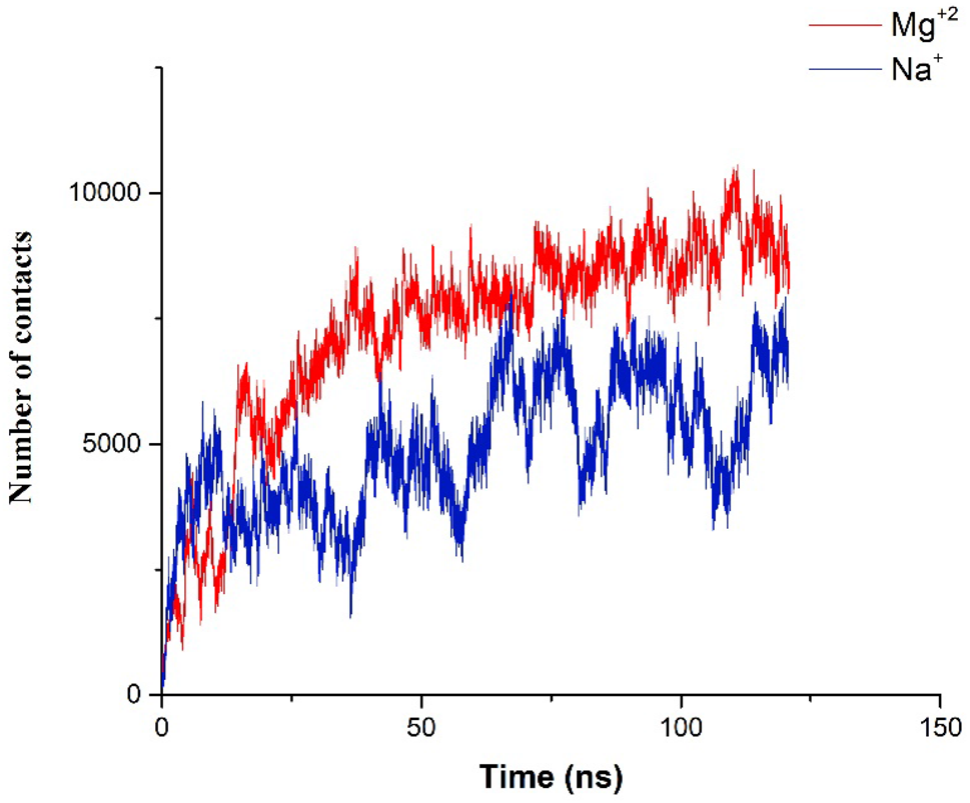
The number of contacts between siRNA and the membrane in the presence of monovalent and divalent cations.

To investigate the stability of siRNA–lipid interaction, the number of contacts between siRNA and lipid was measured. Our findings showed that the number of contacts in the presence of divalent cations significantly increased related to this interaction in the absence of divalent cations Fig. 3. It seems the divalent cations can modulate the interaction between siRNA and head groups of lipids at the membrane surface Fig. 3. In addition, in siRNA–lipid interactions, the unpaired nucleotides at two ends play an essential role and these regions of siRNA could contribute to the formation of the siRNA and lipid interaction^34^. Janas et al. showed that the RNA binding to all phospholipid bilayers is sensitive to ionic strength. These findings suggest that the electrostatic affinity plays an important role in RNA–membrane interaction^53^.

The unpaired ends in siRNA play an important role in different interactions between siRNA and DNA with the membrane^40^. Because of these ends, the siRNA can bind to a neutral membrane and DNA cannot bind to the surface of the membrane^40^. Previous findings showed that divalent cations such as calcium and Mg^2+^ could aid in the formation of interactions between siRNA–lipids^30,34^. However, the molecular details of these interactions are not clear and there are few studies about the siRNA–lipid interaction and the effect of divalent ions such as Mg^2+^ on these interactions. To measure the effect of Mg^2+^ as a divalent cation on the siRNA–lipid system, we carried out molecular dynamics simulations of the siRNA–lipid system in the presence and absence of MgCl_2_. Our results showed that the interaction between siRNA–lipid boosted in the presence of Mg^2+^ cations and formed the stable siRNA lipid interaction. In the presence of Mg^2+^ ions, the distance between siRNA and the center of mass of the membrane decreased and the number of contacts between head groups of lipids and siRNA significantly increased in Figs. 2,3. To investigate the adsorption of the siRNA on the surface of the bilayer, the RDF parameter was measured. To calculate the RDFs, certain atoms, such as the head group of lipids were considered reference atoms. In the following, the distribution of siRNA related to the reference atoms was calculated. Our results indicated that in the presence of Mg^2+^, the mean pick was located at 1.1 nm. This peak is related to the interactions between the siRNA and the phosphate groups of the bilayer. Whereas, in the absence of magnesium ions, the mean pick is located at 2.4 nm Fig. S2**.** Therefore, in the presence of magnesium ions, the siRNA is closer to the bilayer. These events lead to a stable siRNA-bilayer interaction in the presence of magnesium ions. Adamala et al. showed that the magnesium ions could improve the formation of membrane–duplex RNAs and protect RNA molecules from degradation^54^. In addition, Adamala et al. demonstrated that magnesium ions have an important role in the biological activities of RNA molecules. The magnesium ions can bind to RNA molecules and significantly improve the RNA function under cellular conditions^55^.

Previous studies showed that the interaction between the ion and a head group of lipids has an important role in the adsorption of siRNA on the membrane surface^25,56^. Morzy et al. investigated the effect of the environmental parameters on the attachment of dsDNA to the cell membrane. Their results showed that ionic conditions such as MgCl_2_ and the phase of the lipid membrane affect the attachment of dsDNA on the membrane surface, and divalent cations form bridges between nucleic acid and the gel-phase bilayer^35^.

### Distribution of divalent ions in siRNA–bilayer system

**Figure 4:**
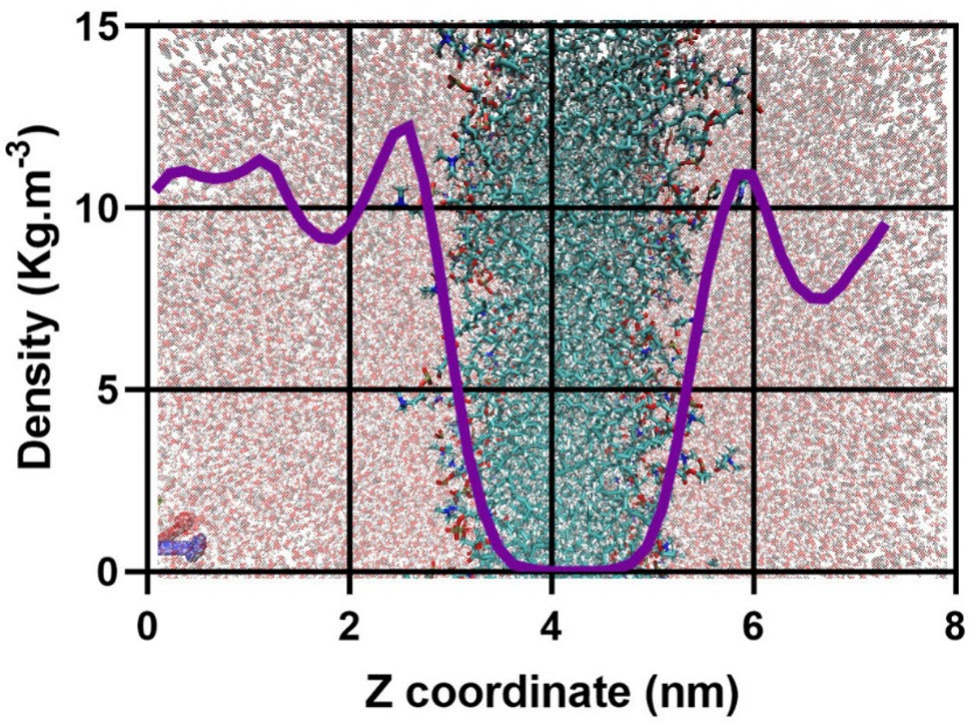
The distribution of divalent cations in the siRNA—membrane system. The purple line is related to the density of Mg ions. In the range of 3–6 nm of z coordinate, the density of Mg ions is around zero because of the hydrophobic region of the membrane.

**Figure 5:**
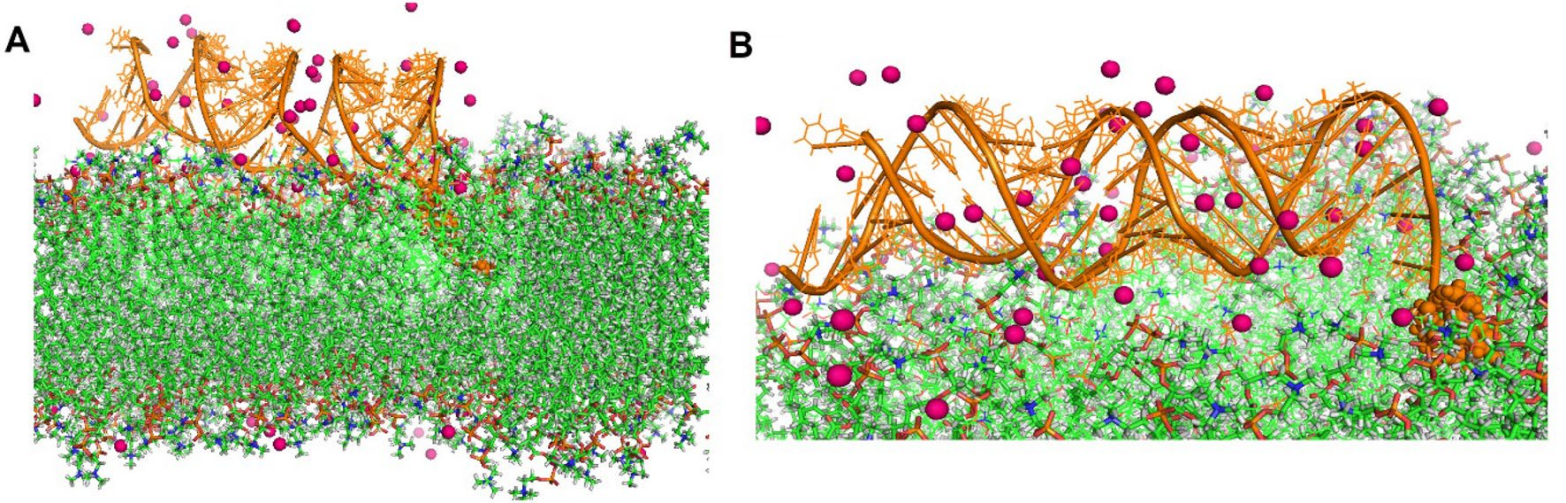
The presentation of the lipid bilayer and siRNA in the presence of Mg^2+^ ions that surrounded it using VMD (**A**) and (**B**) pictures are related to the adsorption of siRNA molecules on the membrane, which is surrounded by Mg^2+^ ions. Many Mg^2+^ ions were located at minor and major grooves of siRNA which can affect the structure of RNA.

The distribution of divalent cations in siRNA – membrane was calculated. Our results showed that the Mg^2+^ ions quickly bound to the surface of the membrane in MD simulation Fig. 4. The distribution of Mg ions at 3–5 nm in the z direction of the box is near zero. This region is related to the hydrophobic part of the bilayer. The highest density of cations is around the surface of the bilayer. By getting away from the surface, the density of cations decreased Fig. 4. These events could modulate the electrostatic interaction between siRNA and the surface of the lipid bilayer. The Mg^2+^ ions bound to the siRNA through MD simulation Fig. 5A,B. The phosphate groups of siRNA with negative charges were suitable sites for binding Mg^2+^ ions on siRNA molecules. Previous studies demonstrated that the Mg^2+^ ions could bind to phosphate groups in two strands of siRNA^25,56,57^. Morzy et al. demonstrated that the monovalent ions are unable to form bridges between siRNA and lipids. It seems the interaction plays a key role in the stability and preservation of the A-form structure in siRNA molecules. The previous finding showed that the cation ions could bind to the surface of the lipid bilayer and siRNA molecules^58^. The binding of divalent ions to the surface bilayer and siRNA leads to the formation of the bridge between siRNA and the lipid bilayer.

### Structure and stability of siRNA molecules

**Figure 6:**
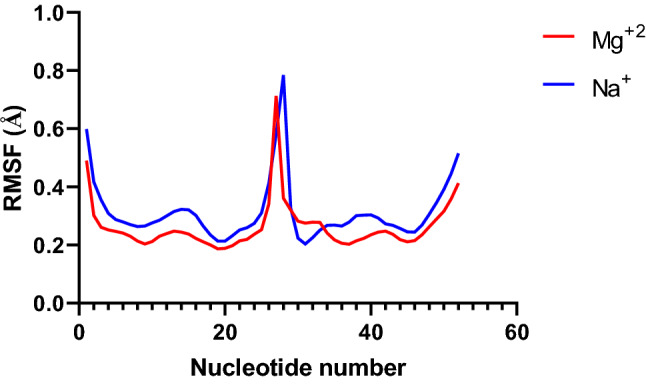
The root mean score fluctuation (RMSF) of siRNA in the presence of Mg^2+^ ions and in the presence of monovalent ions. The siRNA is double stranded. Each chain of the siRNA has 23 nucleotides.

To observe the effect of Mg^2+^ ions on siRNA structure, the minor and major grooves of siRNA as well as the stability and fluctuation of siRNA were analyzed during MD simulation. Our finding showed that in the presence of Mg^2+^ ions, the root mean score fluctuation (RMSF) of siRNA was lower than in the absence of Mg^2+^ ions Fig. 6. It seems the binding of ions to phosphate groups of siRNA can contribute to increasing the stability of siRNA. Schauss et al. reported that the magnesium ion plays an important role in the stability of RNA molecules. Their results showed that the bridges between phosphate groups and magnesium ions in grooves stabilize the tertiary structure of RNA^59^. In addition, the Mg^2+^ ions can be localized in grooves in siRNA and decrease the distance between phosphate groups of two strands. These events can stabilize the A-form structure of siRNA and increase the siRNA stability.

### Physical properties of the bilayer in the siRNA–bilayer system

Physical properties of the cell membrane play an essential role in biological processes. The lateral diffusion of lipids is one of the main factors in the biological processes of the membrane^60,61^. The lipid lateral dynamics for lipids was observed using the mean square displacements (MSD). During MD simulation, in the absence of Mg ions, a linear increase in the mean square displacements was observed. Our findings showed that in the presence of Mg ions, the siRNA can create a stable interaction with the bilayer. These events modulated the decrease in lateral diffusion of lipids Fig. S3. Firstly, siRNA bound to the surface of the bilayer and formed the contacts between the head groups of lipids and siRNA. This event leads to a decrease in the lateral diffusion of the lipids in bilayer Fig. S3. Previous studies reported the nucleic acid–lipid interaction is regulated by both the lipid composition and phase of the bilayer^62^. In addition, our results show that the thickness of the bilayer in the presence and absence of siRNA are 3.6 and 4.1 nm Fig. S4. It seems nucleic acids can change the physical properties of the bilayer, and this interplay influences the attachment and transport across the cell membrane. Kato et al. investigated the effect of the membrane phase on DNA structure and adsorption. Their results showed that DNA molecules were adsorbed on the liquid order phase. Unlike in the liquid disorder phase, DNA molecules cannot attach to the membrane surface^63^. Janas et al. observed the interaction of lipid and RNA molecules using fluorescence and fluorescence resonance energy transfer microscopy. Their results showed that both the structure of RNA and membrane order can modulate the RNA-lipid interaction ^53^. Janas et al. showed that the affinity of RNA for a lipid bilayer was modulated by lipid structure, lipid charge, and ionic strength^64^.

### The role of divalent cations in the attachment of siRNA-cholesterol conjugate on the bilayer surface

Several approaches have been developed to increase the efficiency of siRNA delivery through the cell, such as electric pulse, hydrodynamic injection, and carriers^56,65^. A wide range of carriers exist that help to cross siRNA through the cell membrane and protect it from degradation^66^. The siRNA-cholesterol conjugated is known as the best-conjugated system in siRNA delivery^45^. Despite this, the molecular details of the interaction of siRNA-cholesterol conjugated with the cell membrane and the effect of the ion condition on its efficacy are unknown. Therefore, in this section, we demonstrate the interaction of siRNA-cholesterol modified and membrane and the effect of ion condition on its attachment.

Our findings demonstrated that in the presence of divalent cations, the electrostatic repulsion between siRNA and head groups of lipids is formed. The adsorption of siRNA on the bilayer surface is regulated by two parameters: the hydrophobic interactions between the cholesterol tag modified siRNA and the bilayer and electrostatic interactions between siRNA and lipid head groups. Our results showed that the affinity of siRNA to the bilayer depends on the ionic concentration. It seems the ionic concentration plays an essential role in siRNA adsorption on the bilayer surface and regulates the hydrophobic interaction between the cholesterol tag and the bilayer surface.

**Figure 7:**
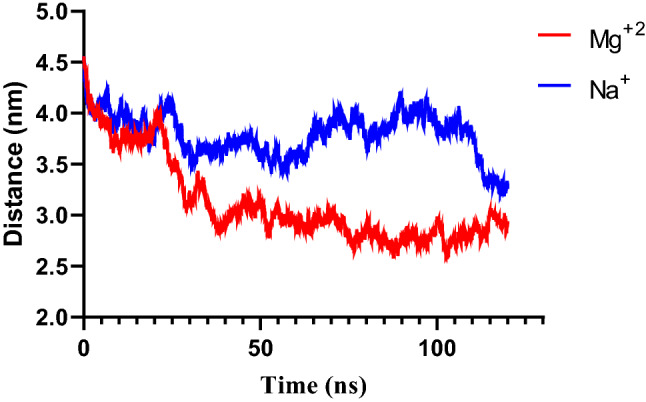
The distance between the center of mass of siRNA modified by cholesterol and head group lipids during the MD simulation.

In this section, the interaction and translocation of siRNA and lipid bilayer were observed. To observe the affinity of siRNA on the bilayer surface, the distance between the COM (center of mass) of end groups of siRNA modified by cholesterol and head group lipids was measured during the MD simulation and plotted in Fig. 7. The distances in the presence and absence of Mg^2+^ ions are 3.0 and 3.8 nm Fig. 7. Our findings show that in the presence of Mg^2+^ ions, the distance significantly decreases. It seems that, in the presence of Mg^2+^ ions, the complex siRNA and lipid bilayer are more stable. Indeed, the divalent cations contribute to the formation of the bridges between head groups of lipids and siRNA, and in the following, the electrostatic interaction between siRNA and head groups of the membrane increases^53,67^.

**Figure 8:**
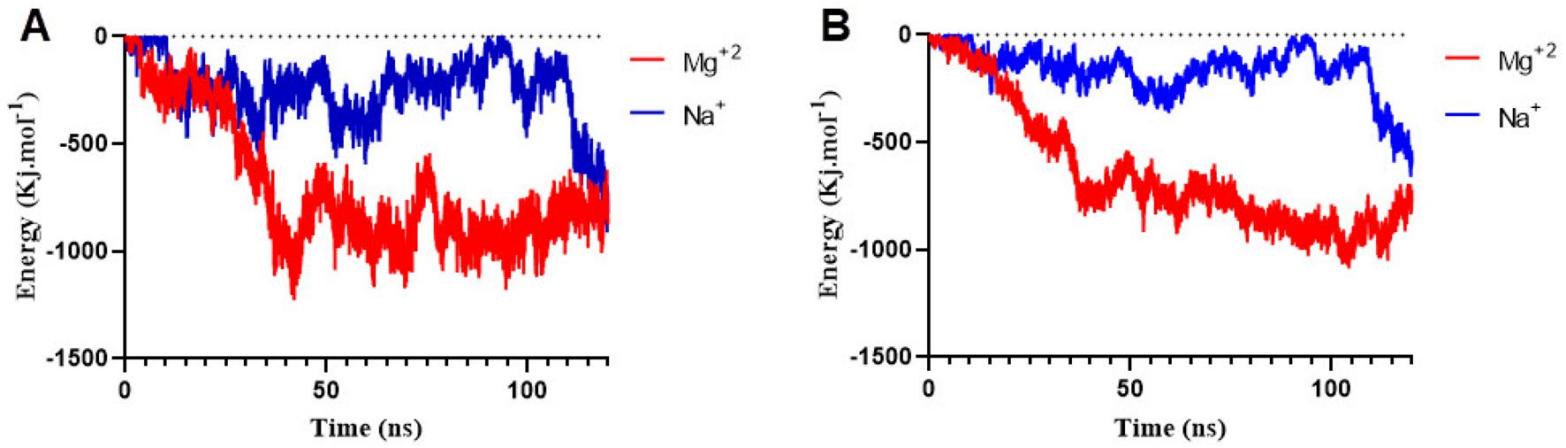
(**A**) The electrostatic and (**B**) vdW energy between the siRNA-cholesterol-conjugate system and lipid bilayer in the presence of Mg^2+^ ions and in the presence of monovalent ions.

The hydrophobic interaction between cholesterol-tagged siRNA and membrane lipids is regulated by cation ions^35^. In order to investigate the thermodynamic properties of adsorption of siRNA on the bilayer surface, the electrostatic and vdW energies between siRNA and the lipid bilayer were computed. In the presence of Mg^2+^ ions, siRNA has a stronger interaction with bilayer head groups and facilitated the interaction of cholesterol tagged with bilayer Fig. 8A,B. In the presence of Mg ions, the electrostatic energy between the bilayer and siRNA was around – 960 kj/mol and the vdW energy siRNA-bilayer was – 940 kj/mol, whereas the electrostatic energy between the bilayer and siRNA was around – 270 kj/mol and the vdW energy was – 170 kj/mol. In presence of Mg^2+^ ions, the electrostatic and vdW energies between siRNA–lipid were threefold related to these energies in the presence of sodium ions Fig. 8. Previous research has shown that monovalent cations cannot form bridges between siRNA phosphate groups and lipid head groups^37^. Therefore, the electrostatic interactions of siRNA-lipid in the presence of magnesium ions were higher than those of monovalent ions. In the presence of Mg^2+^ ions, the interaction between siRNA and lipid bilayer is facilitated, and the electrostatic interaction between siRNA and lipid head groups is higher than in the absence of Mg^2+^ ions. The electrostatic interactions play an essential role in siRNA adsorption on the bilayer surface Fig. 8.

**Figure 9:**
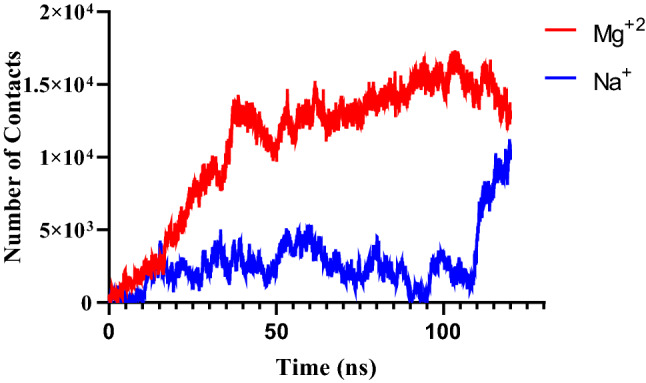
The number of contacts between siRNA-cholesterol-conjugate system–lipid bilayer.

In addition, in order to gain further insight into the affinity of siRNA on the bilayer surface, the number of contacts between siRNA-lipid was calculated. Results show that in the presence of Mg^2+^ ions, the number of contacts between siRNA–head group of lipids is higher than in the absence of Mg^2+^ ions Fig. 9. Our results show that in the presence of Mg^2+^ ions, the contacts between siRNA and the head group of lipids rapidly increased, which is higher than siRNA-Lipid without Mg^2+^ ions^68^. The curve of contacts between siRNA-bilayer in the presence of sodium ions was unstable and in most of the simulation time was around 3000. In the presence of Mg^2+^ ions, the number of contacts between the siRNA-bilayer was around 15,000. In fact, the number of contacts siRNA-bilayer in presence of Mg^2+^ ions is five time higher than in presence of sodium ions Fig. 9. These findings show that the Mg^2+^ ions can modulate the attachment of siRNA to the lipid bilayer.

The interaction of Mg^2+^ ions on the membrane surface and siRNA is a bridge to modulate the siRNA lipid interaction. Whereas, the monovalent cations are unable to form bridges between phosphate groups of siRNA and head groups of lipid. Our results demonstrated that in the presence of divalent ions, the number of contacts between siRNA and the membrane significantly increased and the distance between siRNA and the membrane decreased. It seems the attractive interactions between siRNA and the bilayer are a phenomenon in which divalent ions such as Mg^2+^ ions could be modulated.

## Conclusion

The delivery of siRNA into cells and translocation into cytoplasm are the main challenges in the clinical application of siRNA. The understanding of the mechanism of the siRNA and zwitterion membrane and how ionic conditions can affect this mechanism at the molecular level will provide important insight into the delivery of siRNA into cells and the translocation of siRNA into the cytosol. The ionic concentration has a critical role in the attachment of siRNA to the cell membrane. We showed how the divalent ions (Mg^2+^ ions) could affect the delivery of siRNA through the membrane and the interaction of siRNA with the membrane surface.

The unpaired nucleotides at the two ends are an important parameter in the adsorption of siRNA on the membrane. It seems the unpaired nucleotides at the terminals of siRNA are an anchor which attaches to the bilayer surface. Therefore, the unpaired nucleotides at the terminal of siRNA are critical in the siRNA attachment. Because of their absence in the DNA, the adsorption of DNA and siRNA on the bilayer surface is different. The Mg^2+^ ions can bind to head groups of lipids which changes the physical properties of the membrane surface. Our results demonstrated that the ionic concentration could be key to the modulation of the interactions of siRNA and the membrane surface. In addition, the divalent cations could affect the structural properties of siRNA and its attachment to the membrane surface. The cations ions can be localized at minor and major grooves and stabilize the A-form of siRNA. We showed that divalent cations can regulate the bridging-driven membrane attachment, and it seems this modulating effect of cations can be used in designing biomimetic devices.

A wide range of carriers exist that help to cross siRNA through the cell membrane and protect it from degradation. The siRNA-cholesterol conjugated is known as the best-conjugated system in siRNA delivery. Despite this, the molecular details of the interaction of siRNA-cholesterol conjugated with the cell membrane are unknown. In our study, we showed that in the presence of divalent ions, the siRNA has higher adsorption on the membrane surface. We showed that the electrostatic interaction between membrane and siRNA cannot be facilitated by cholesterol conjugated only. Cations are essential to create coulomb repulsion and enable membrane attachment. It seems the divalent cations required for the formation of the stable attachment of siRNA modified by cholesterol on the bilayer. Due to the coronavirus pandemic outbreak, these results may shed light on the new approach for treating these diseases and their molecular details.

## Supplementary Information

Supplementary Figures.

## Data Availability

All data generated or analyzed during this study is included in this published article. The raw data could be sent upon request.
