# Supplementary material for: Investigation of the ionic conditions in SiRNA-mediated delivery through its carriers in the cell membrane: a molecular dynamic simulation
Source: Sci Rep. 2022 Oct 20;12:17520. doi: 10.1038/s41598-022-22509-1 (PMC9582388; doi:10.1038/s41598-022-22509-1)
Supplement: Supplementary file 1 — Supplementary Figures. [file 41598_2022_22509_MOESM1_ESM.docx]

**Supporting information**

Investigation of the ionic conditions in SiRNA-mediated delivery through its carriers in the cell membrane: A molecular dynamic simulation

Mohammad Hasan Darvishi ^§*^, Abdollah Allahverdi ^†^, Hadi Hashemzadeh ^†^, Hamid Reza Javadi ^§^

§ *Nanobiotechnology Research Center, Baqiyatallah University of Medical Sciences, Tehran, Iran*

† *Department of Biophysics, Faculty of Biological Science, Tarbiat Modares University, 14115-154 Tehran, Iran*

* **Corresponding Author:**

E-mail: [darvishi@alumnus.tums.ac.ir](mailto:darvishi@alumnus.tums.ac.ir)


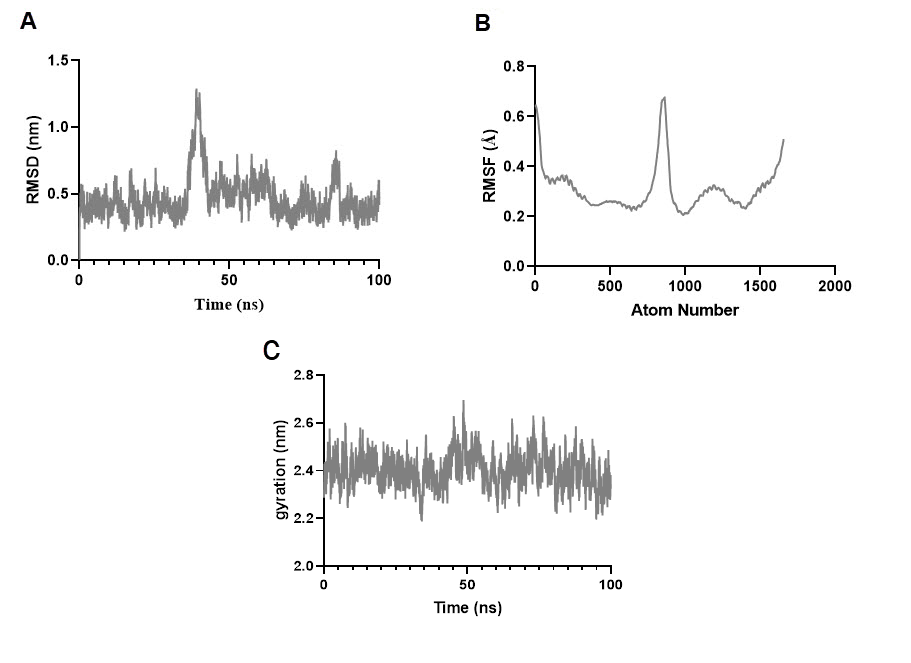


**S1.** The Root mean score deviation (RMSD) and Root mean score fluctuation (RMSF) and gyration parameter of siRNA during MD simulation


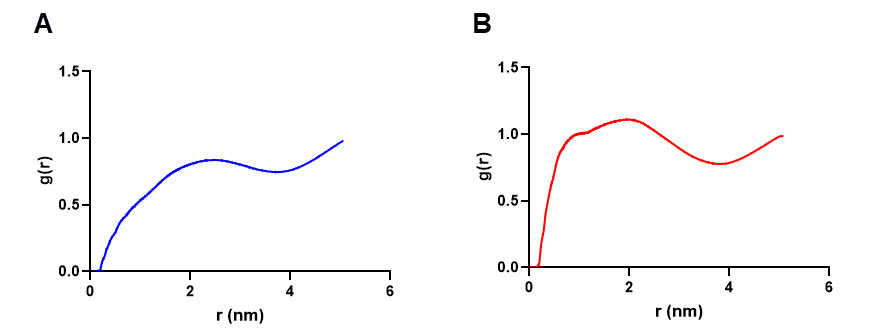


**S2**. Radial distribution function (RDF) of the center of mass of the siRNA molecule with respect to the head group of lipids in the presence of monovalent cation (A) and divalent cation (B)


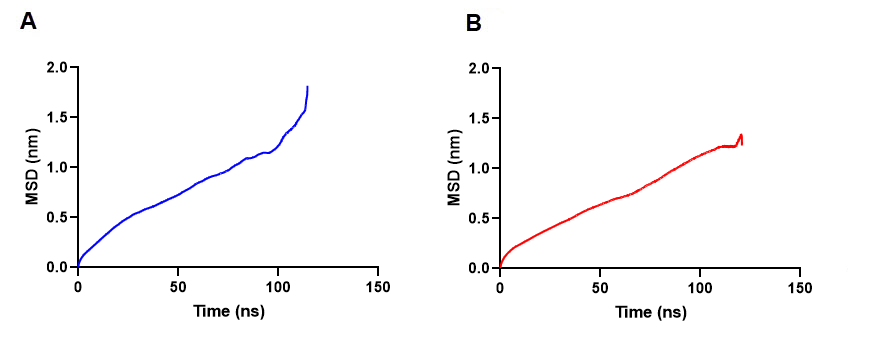


**S3.** The MSD of bilayer in the absence (A) and in the presence of magnesium ions (B)

**S4**. Thickness of bilayer in the presence of magnesium and sodium ions

**S5.** The distance between the end of siRNA and head group lipids during the MD simulation

**S6**. The electrostatic and von der Waals (vdW) energy between siRNA and lipid bilayer in the presence of Mg^+2^  ions and in presence of monovalent ions

| **SYSTEM** | **Electrostatic Energy** (Kj/mol) | **vdW energy**  (Kj/mol) |
| --- | --- | --- |
| RNA-Membrane | -463.447 | -690.099 |
| RNA-Membrane / Mg^+2^ ions | -921.715 | -506.397 |


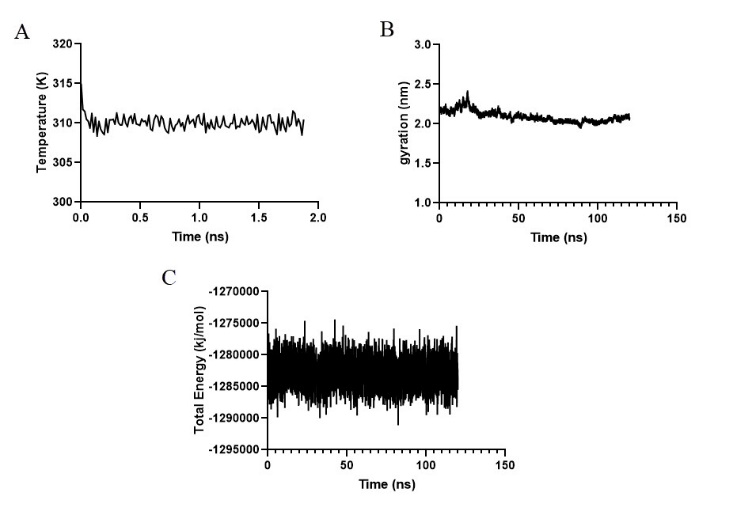


**S7.** (A) The temperature of system during the equilibration step of MD simulation. (B) The gyration of system during MD simulation, (C) the total energy of system during MD simulation.
